# Rare Copy Number Variations in a Chinese Cohort of Autism Spectrum Disorder

**DOI:** 10.3389/fgene.2018.00665

**Published:** 2018-12-18

**Authors:** Yanjie Fan, Xiujuan Du, Xin Liu, Lili Wang, Fei Li, Yongguo Yu

**Affiliations:** ^1^Shanghai Institute for Pediatric Research, Xinhua Hospital Affiliated to Shanghai Jiao Tong University School of Medicine, Shanghai, China; ^2^Department of Developmental and Behavioral Pediatrics, Department of Child Primary Care, Brain and Behavioral Research Unit of Shanghai Institute for Pediatric Research & MOE-Shanghai Key Laboratory for Children's Environmental Health, Xinhua Hospital Affiliated to Shanghai Jiao Tong University School of Medicine, Shanghai, China; ^3^Department of Developmental and Behavioral Pediatrics, Department of Child Primary Care, Xinhua Hospital Affiliated to Shanghai Jiao Tong University School of Medicine, Shanghai, China

**Keywords:** autism spectrum disorder, rare copy number variations, chromosomal microarray, LoF-intolerant genes, *RIMS2*

## Abstract

Autism spectrum disorder (ASD) is heterogeneous in symptom and etiology. Rare copy number variations (CNVs) are important genetic factors contributing to ASD. Currently chromosomal microarray (CMA) detecting CNVs is recommended as a first-tier diagnostic assay, largely based on research in North America and Europe. The feature of rare CNVs has not been well characterized in ASD cohorts from non-European ancestry. In this study, high resolution CMA was utilized to investigate rare CNVs in a Chinese cohort of ASD (*n* = 401, including 177 mildly/moderately and 224 severely affected individuals), together with an ancestry-matched control cohort (*n* = 197). Diagnostic yield was about 4.2%, with 17 clinically significant CNVs identified in ASD individuals, of which 12 CNVs overlapped with recurrent autism risk loci or genes. Autosomal rare CNV burden analysis showed an overrepresentation of rare loss events in ASD cohort, whereas the rate of rare gain events correlated with the phenotypic severity. Further analysis showed rare losses disrupting genes highly intolerant of loss-of-function variants were enriched in the ASD cohort. Among these highly constrained genes disrupted by rare losses, *RIMS2* is a promising candidate contributing to ASD risk. This pilot study evaluated clinical utility of CMA and the feature of rare CNVs in Chinese ASD, with candidate genes identified as potential risk factors.

## Introduction

Autism spectrum disorder (ASD) is characterized by persistent deficits in social communication and restricted, repetitive pattern of behaviors (Lai et al., 2014). The manifestation of ASD spans a broad range of symptoms and severity (Lai et al., 2014). This phenotypic diversity coincides with heterogeneous genetic etiology—known genetic causes of ASD include aneuploidy, copy number variations (CNVs), and single nucleotide variations (De Rubeis and Buxbaum, 2015).

Multiple lines of evidence support rare CNVs as an important type of genetic factors contributing to autism risk (Schaefer et al., 2013), and currently chromosomal microarray (CMA) detecting CNVs is recommended as a first-tier diagnostic assay for ASD (Miller et al., 2010). However, most of these evidence come from studies in North America and Europe (Shen et al., 2010; Schaefer et al., 2013; Tammimies et al., 2015). Research on rare CNVs in ASD from non-European ancestry is limited but necessary, considering the substantial difference of CNV distribution and pattern due to ethnical diversity (Park et al., 2010; Manrai et al., 2016). For Chinese population, only three studies so far have examined the yield of CMA in ASD (probands from Northern China, Taiwan and Hong Kong, respectively; Gazzellone et al., 2014; Yin et al., 2016; Mak et al., 2017), which primarily focused on the clinical utility, necessitating further work to characterize the general feature and burden of rare CNVs. Besides being short of ethnical diversity, published CNV studies are largely from ASD cohorts with varying degrees of severity. The correlation between rare CNV burden and symptom severity has not been investigated yet. Dissecting the heterogeneity of severity is a critical step to understand the genetic architecture of ASD.

Interrogating the genic content of rare CNVs is another aspect to gain insights of ASD etiology, as candidate genes can be discovered in rare CNV regions. Given the strong selective pressure on neurodevelopmental disorders (Kosmicki et al., 2017), genes intolerant of loss-of-function (LoF) variants are prioritized candidates. Among the 86 genes curated as high-risk factors by SFARI (Simons Foundation Autism Research Initiative, https://gene.sfari.org), 63 were LoF-intolerant genes (based on pLi score>0.99 in Exome Aggregate Consortium; Ruderfer et al., 2016). Interrogating these evolutionally constrained genes in rare CNVs is a rational approach of candidate search.

In this study, we investigated rare CNVs in a well-characterized Chinese ASD cohort (*n* = 401), including 177 mildly affected and 224 severely affected individuals, together with an ancestry-matched control cohort (*n* = 197). Three aims of this study are: 1. To evaluate the diagnostic yield of CMA in Chinese ASD individuals; 2. To examine the rare CNV burden between mildly and severely affected subgroups; 3. To identify candidate risk genes based on rare CNVs disrupting those genes extremely intolerant of LoF variants.

## Materials and Methods

### Sample Selection

Four hundred and one Chinese individuals diagnosed of ASD were recruited during July 2014 to December 2017 from the Developmental and Behavioral Clinic at Xinhua Hospital and Shanghai Children's Medical Center. Diagnostic and Statistical Manual of Mental Disorders, Fifth Edition (DMS-5) (American Psychiatric Association, 2013), the Autism Diagnostic Observation Schedule (ADOS) (Lord, 2002), and Childhood Autism Rating Scale (CARS) (Schopler et al., 1986) were used. The ASD cohort consisted of 335 males and 66 females, with age ranged from 1 year 5 months to 17 years old. The severity categorization was based on CARS score −30–37 defined as mildly/moderately affected (Mild group), and 37–60 defined as severely affected (Severe group). Full list of this ASD cohort was included in Supplementary Table 1. The control cohort consisted of 123 males and 85 females of Chinese with no ASD or other major anomalies.

This study was carried out in accordance with the recommendations of national guidelines on research involving human subjects in China with written informed consent. All subjects gave written informed consent in accordance with the Declaration of Helsinki. For participants under 16, written informed consent was obtained from the parents of the participants. The protocol was approved by the Ethics Committee of Xinhua hospital.

### CMA and Data Analysis

Genomic DNA was extracted from peripheral blood of participants. Affymetrix CytoScan HD array (average probe spacing 1,148bp) was utilized to detect genomic CNVs following the manufacturer's guide (Thermo-Fisher Scientific, United States). Array results were analyzed by Chromosome Analysis Suite software with streamlined CNV calling workflow (Thermo-Fisher Scientific, USA). CYCHP files generated were used for summarizing chromosomal aberrations. Size threshold was set to 100 kb (with >25 probes), a relatively stringent criterion to ensure high confidence CNV calling.

### Rare CNV Burden Analysis

Population control data was obtained from 2,691 phenotypically normal controls analyzed by the same CMA platform (dataset offered by Affymetrix) and from Database of Genomic Variants (http://dgv.tcag.ca/dgv/app/home) (MacDonald et al., 2014). One percent frequency threshold (defined as >50% overlap of length) was applied to retain only rare CNVs. Burden analysis for rare CNVs was performed using PLINK v1.07 and scripts developed in house. Due to the imbalance of gender in the ASD cohort, only autosomal rare CNV burden was analyzed in this study. Three aspects of rare CNV burden were evaluated: the rate (the number of rare CNV events per individual), the CNV size, and the proportion of individuals harboring at least one event. *P*-values were estimated by permutation function in PLINK (http://pngu.mgh.harvard.edu/purcell/plink/) (MacDonald et al., 2014). The setting of one-sided, 100,000 permutations was used for these comparisons—ASD vs. control, and Severe vs. Mild.

### Regions of Interest

The chromosomal regions of known ASD loci were based on the summary by Pinto et al. including well-established ASD loci with multiple lines of evidence (Pinto et al., 2014). The list of high-risk ASD genes with strong evidence was quoted from the curated SFARI Gene database (https://gene.sfari.org/, “category 1–high confidence” and “category 2–strong candidate”). LoF-intolerant gene list was generated based on the pLi score in Exome Aggregation Consortium (http://exac.broadinstitute.org/, genes with pLi>0.99 were included) (Lek et al., 2016). These lists of genes were included in Supplementary Table 2.

CNVs overlapping with chromosomal regions of known ASD loci (>80% overlap, and of the corresponding type of deletion or duplication) were considered clinically relevant and counted as “with ASD loci” in burden analysis. In the analysis of potentially disruptive events of LoF-intolerant genes, any loss events intersecting the genic regions were counted, while gain events were counted only when starting or stopping within the genic regions (resulting in partially duplicated genes).

## Results

### Diagnostic Yield of CMA in Chinese ASD

Based on the American College of Medical Genetics and Genomics (ACMG) guideline of CNV interpretation (Kearney et al., 2011), 17 out of 405 individuals in our ASD cohort were found to harbor pathogenic CNVs. These CNVs included: (1) 6 CNVs in the regions of 8p23.3p23.1, 10q11.2, 4q31.21q33, 3p14.1, and 17p12; (2) 10 CNVs with at least 80% overlap of known ASD loci; and (3) 2 CNVs involving high-risk ASD genes, among which the *TAOK2*-relevant CNV also resided in the known 16p11.2 ASD loci. These CNVs were summarized in **Table 2** (see “2.4 Region of interest” for details of known ASD loci and high-risk ASD genes). This resulted in approximately 4.2% diagnostic yield of CMA in the ASD cohort.

### Rare CNV Burden in ASD vs. Control and the Correlation With Severity

The occurrence of rare CNV events was summarized in Table 1, stratified by CNV type and size. Only autosomal CNVs were analyzed in this study, considering the gender bias in the ASD cohort. When taking all the CNVs above 100 kb into consideration, the average occurrence of rare loss event was 0.369 per person in the ASD cohort, significantly higher than the occurrence rate of 0.259 per person in the control cohort (R_ASD_/R_Control_ = 1.43, *p* = 0.021, one-sided, 100,000 permutations). The rate of rare loss events at all size ranges was nominally higher in the ASD cohort, but no statistical difference was reached at particular size range. For rare gain events, no significant difference of the occurrence rate was found between the ASD and control cohort (R_ASD_/R_Control_ = 0.76, *p* = 0.996).

**Table 1:** The occurrence of rare CNVs by type, size and regions of interest.

	**Type**	**Size**	**Control** ***n* = 197**	**ASD** ***n* = 401**	**R_**ASD**_/** **R_**control**_**	***P*** **(ASD vs.** **Control)**	**Mild ASD** ***n* = 177**	**Severe ASD** ***n* = 224**	**R_**Severe**_/** **R_**Mild**_**	***P* (Severe vs Mild)**
Rare CNVs by type and size	Loss	All	51	148	**1.43**	**0.021**	72	76	0.83	0.861
		>3M	0	1	∞	1.000	1	0	0.00	1.000
		1M-3M	1	11	5.40	0.072	7	4	0.45	0.931
		400k-1M	4	13	1.60	0.292	9	4	0.35	0.984
		100k-400k	46	123	1.31	0.070	55	68	0.98	0.583
	Gain	All	176	272	0.76	0.996	97	175	**1.43**	**0.003**
		>3M	0	6	∞	0.090	1	5	3.95	0.173
		1M-3M	7	10	0.70	0.828	3	7	1.84	0.308
		400k-1M	23	50	1.07	0.451	19	31	1.29	0.234
		100k-400k	146	206	0.69	0.999	74	132	**1.41**	**0.010**
w/ASD loci	Loss	All	0	6	∞	0.088	4	2	0.40	0.939
	Gain	All	0	4	∞	0.060	0	4	∞	0.096
w/HiRisk genes	Loss	All	0	2	∞	0.602	1	1	0.79	1.000
	Gain	All	4	2	0.25	0.917	0	2	∞	0.310
w/LoF-I genes	Loss	All	2	12	2.95	0.070	5	7	1.11	0.405
	Gain	All	16	32	0.98	0.772	10	22	1.74	0.087

*R, rate of CNV (number of events per individual); HiRisk genes, high-risk ASD genes; LoF-I genes, LoF-intolerant genes. See “2.4 Regions of interest” for details. P < 0.05 was displayed in bold*.

In the comparison between ASD individuals categorized by severity, rare gain events occurred at a higher rate in the severely affected individuals (right panel of Table 1, R_Severe_/R_Mild_ = 1.43, *p* = 0.003), and this difference was significant at the small size range of 100–400 kb (R_Severe_/R_Mild_ = 1.41, *p* = 0.010). No correlation between the occurrence rate of rare loss events and ASD severity was found (“All” size range, R_Severe_/R_Mild_ = 0.83, *p* = 0.861).

Besides the occurrence rate, we also analyzed two other parameters of burden, including CNV size and the proportion of individuals harboring at least one rare CNV event. However, no significant difference in these two measures was found, either in the comparison of “ASD vs. control” or in “Severe vs. Mild” (data not shown).

Taken together, the difference of CNV burden between ASD and control was mainly in the rate of rare loss events, while within the ASD cohort, the severity correlated with the rate of rare gain events.

### Rare CNVs in Regions of Interest–Recurrent ASD Loci and High-Risk ASD Genes

A total of 10 rare CNVs (6 losses and 4 gains) overlapped with known recurrent ASD loci (see Supplementary Table 2 for the list of chromosomal locations). Among these loci, 15q11q13 duplications and 22q11.2 deletions were recurrently found in the ASD cohort (Table 2). Heterozygous loss of two high-risk ASD genes—*NRXN1* and *TAOK2*—were found in two ASD individuals.

**Table 2:** Rare CNVs with clinical significance, in known ASD loci or intersected with high-risk genes.

	**Loci/Gene**	**CNV(hg19)**	**Size(kb)**	**Type**	**ID**	**Severity**	**Interpretation**
	8p23.3p23.1	chr8:158049-10137194	9,979	Loss	ASD425	Mild	Pathogenic
	10q11.2	chr10:46206776-51812795	5,606	Gain	ASD106	Severe	Pathogenic
	4q31.21q33	chr4:145149738-170414221	25,264	Gain	ASD215	Mild	Pathogenic
	3p14.1	chr3:66066409-69273190	3,207	Gain	ASD343	Severe	Pathogenic
	17p12	chr17:14087934-15484859	1,397	Loss	ASD445	Severe	Pathogenic
		chr17:14087934-15491532	1,404	Loss	ASD381	Mild	Pathogenic
In known ASD Loci	1q21.1	chr1:146106724-147926347	1,820	Loss	ASD227	Mild	Pathogenic
	7q11.23	chr7:72624167-74136633	1,512	Loss	ASD309	Severe	Pathogenic
	16p13.11	chr16:15449697-16294705	845	Loss	ASD443	Mild	Pathogenic
	16p11.2	chr16:29580021-30178406	598	Loss	ASD386	Severe	Pathogenic
	22q11.2	chr22:18644791-19899146	1,254	Loss	ASD419	Mild	Pathogenic
		chr22:18916843-21798907	2,882	Loss	ASD017	Mild	Pathogenic
	1q21.1	chr1:146498360-147399145	900	Gain	ASD278	Severe	Pathogenic
	15q11q13	chr15:22770422-28545355	5,775	Gain	ASD050	Severe	Pathogenic
		chr15:23284500-28534245	5,250	Gain	ASD061	Severe	Pathogenic
		chr15:22770422-28560664	5,790	Gain	ASD317	Severe	Pathogenic
With high-risk genes	*NRXN1*	chr2:51057960-51433041	375	Loss	ASD287	Mild	Pathogenic
	*TAOK2*	chr16:29580021-30178406	598	Loss	ASD386	Severe	Pathogenic
	*CNTNAP2*	chr7:144519741-145950454	1,431	Gain	ASD406	Severe	Uncertain
	*ADNP*	chr20:49531228-49685636	154	Gain	ASD094	Severe	Uncertain

Rare loss events involving well-known ASD loci and high-risk ASD genes were not found in control. However, the potentially disruptive gain events were found in the control cohort, at even higher rate than in the ASD cohort (lower left part of Table 1). Between the Mild and Severe groups, no significant difference of rare CNV events overlapping with known loci/genes was observed (lower right part of Table 1).

### Genes Intolerant of LoF Variants Intersected by Rare CNVs in the ASD Cohort

Genes intolerant of LoF variants were prioritized candidates of ASD risk factors (see “2.1 Regions of interest” for details of gene list). Rare CNVs identified in the ASD cohort were interrogated for potential disruption of these evolutionally constrained genes. The occurrence of loss events and potentially disruptive gain events (when starting or stopping within genic region, resulting in partially duplicated gene) in genes intolerant of LoF variants was summarized in Tables 1, 3. Loss events intersecting these constrained genes were enriched in ASD cohorts (2.95 times higher rate, Table 1), but potentially disruptive gain events were dispersed in control and ASD. *RIMS2, PTPRT, FRMD4A, HSPA14*, and *CCZ1* were intersected by loss events (Table 3), and a total of 24 LoF-intolerant genes were intersected by potentially disruptive gain events only in the ASD cohort (Supplementary Table 3). Of particular, RIMS2 was found in two rare CNVs (one loss and one gain) in two independent ASD patients, and both of the affected individuals presented severe symptom (Table 3).

**Table 3:** Novel candidate genes intersected by rare CNVs in this study.

**Gene**	**Full name**	**CNV location (hg19)**	**Size(kb)**	**Type**	**DGV freq**	**ID**	**Severity**
*RIMS2*	Regulating synaptic membrane exocytosis 2	chr8:105008757-105127783	119	Loss	0.000169	ASD380	Severe
		chr8:104218356-104552138	334	Gain	0	ASD024	Severe
*PTPRT*	Protein tyrosine phosphatase, receptor type T	chr20:41153385-41326127	173	Loss	0	ASD114	Severe
*FRMD4A*	FERM domain containing 4A	chr10:13219451-15005442	1,786	Loss	0	ASD265	Severe
*HSPA14*	Heat shock protein 90 beta family member 1	chr10:13219451-15005442	1,786	Loss	0	ASD265	Severe
*CCZ1*	Vacuolar protein trafficking and biogenesis associated	chr7:5838735-5982759	144	Loss	0.000676	ASD289	Severe

*DGV freq: the population frequency of respective CNV in Database of Genomic Variants (http://dgv.tcag.ca/dgv/app/home)*.

## Discussion

### Diagnostic Yield of CMA Can Be Affected by Heterogeneity of the ASD Cohort

The diagnostic yield of CMA in this study was 4.2%, as clinically significant CNVs were identified in 17 out of 405 ASD individuals. This yield is slightly lower than majority of ASD studies based on cohorts of European ancestry, which reported a diagnostic rate of 5–10% (Shen et al., 2010; Schaefer et al., 2013). In three published studies on Chinese ASD, the diagnostic rate was reported to be 8.6, 5.1, and 3.5% in cohorts with sample size of 104, 335, and 228, respectively (Gazzellone et al., 2014; Yin et al., 2016; Mak et al., 2017). Besides the potential bias in patient origin and CNV analysis, the difference of diagnostic yields could be attributed to the presence of comorbidity in the cohort. When CMA was performed in ASD patients with comorbid intellectual disability, microcephaly or other congenital anomalies in our center, the yield increased to 15% (Fan et al., 2018). Affected individuals in this study were relatively “pure”–over 95% of the ASD cohort were free of major systemic anomalies. The 4.2% diagnostic yield found in this study is exactly same as the finding in “essential group” of ASD by Tamminies et al., who found the yield increased drastically from 4.2 to 24.5% in the “complex group” with co-presence of morphological anomalies (Tammimies et al., 2015).

### Rare Gain Burden Was Implicated to Correlate With the Phenotypic Severity

Our pilot study on rare CNV burden in ASD of Chinese ancestry suggested increased occurrence of rare loss events in the ASD cohort. This is different from the prior burden analysis on large European cohort showing rare “genic” losses and gains were overrepresented but not the overall occurrence (Pinto et al., 2010). Due to the small sample size in this study, replication study on larger Chinese cohort is necessary to ascertain if CNV burden is influenced by ethnicity.

Our finding also implied higher burden of rare gains in the severe ASD than the mild. Though not exactly the same way, a similar observation that rare gains influenced the phenotypic outcome in ASD was reported, and the authors found the burden of duplications, but not deletions, correlated with the severity score (Girirajan et al., 2013). Our results were in agreement with the finding by Girirajan et al., but replications on larger datasets are warranted.

### Potentially Disruptive CNV Events Were Found in Genes Intolerant of LoF Variants

Rare loss events disrupting genes extremely intolerant of LoF variants were found to be enriched in the ASD cohort, while rare gain events did not show such enrichment. One explanation is that the impact of deletions (loss) on gene function is relatively definite, while partial duplications (gain events counted as “potentially disruptive” in this study) may not have deleterious impact on the interested gene. This may also explain a nominally higher incidence of rare gains intersecting high-risk ASD genes were found in the control cohort (Table 1).

LoF-intolerant genes disrupted by rare loss events in ASD are prioritized candidates in this study. Among the five genes intersected by rare loss events, *RIMS2* was also intersected by a rare gain found in another individual of the ASD cohort. No study so far has reported the association of *RIMS2* with genetic disorders, but its homologues *RIMS3* and *RIMS4* were implicated autism risk factors (Kumar et al., 2010; Leblond et al., 2018). *RIMS2* codes for a presynaptic protein regulating synaptic membrane exocytosis, and mediates neurotransmitter release during short- and long-term synaptic plasticity (Kaeser and Südhof, 2005). Given the well-established role of synaptic plasticity in ASD etiology (Bourgeron, 2015), genetic variants of *RIMS2* could affect the synaptic regulation and confer risk to ASD.

## Conclusion

This study investigated rare CNVs in a Chinese ASD cohort. The diagnostic yield of CMA was 4.2%, and CNV burden analysis suggested overrepresentation of rare losses in ASD, whereas the symptom severity correlated with rare gain burden. Additionally, rare losses intersecting LoF-intolerant genes were enriched in ASD. The CNV burden and potential candidates implicated in this pilot study should be validated in larger ASD cohorts for definite clues of genetic etiology.

## Data Availability Statement

The CNVs in this study can be found in the LOVD database (https://databases.lovd.nl/shared/individuals), with accession numbers #00181115 to #00181139.

## Author Contributions

YF and XD performed the data analysis and drafted the manuscript. XD, XL, and FL collected the clinical information and performed the psychiatric diagnostic evaluation. LW performed the experiments of chromosomal microarray. YF, FL, and YY designed and supervised the study.

### Conflict of Interest Statement

The authors declare that the research was conducted in the absence of any commercial or financial relationships that could be construed as a potential conflict of interest.
